# NK cells from Men Who Have Sex with Men at high risk for HIV-1 infection exhibit higher effector capacity

**DOI:** 10.1038/s41598-023-44054-1

**Published:** 2023-10-05

**Authors:** Daniel S. Rincón, Lizdany Flórez-Álvarez, Natalia A. Taborda, Juan C. Hernandez, María T. Rugeles, Wildeman Zapata-Builes

**Affiliations:** 1 Grupo Inmunovirología, Facultad de Medicina, Universidad de Antioquia UdeA, Medellín, 050010 Colombia; 2 Grupo Infettare, Facultad de Medicina, Universidad Cooperativa de Colombia, Medellín, 050016 Colombia; 3 Corporación Universitaria Remington, Medellín, 050012 Colombia

**Keywords:** Infectious diseases, Infectious diseases, Diseases

## Abstract

Despite being under constant exposure to HIV-1, some individuals do not show serological or clinical evidence of infection and are known as HESN (HIV-Exposed Seronegative). Multiple studies in different HESN cohorts have linked the NK cells as a correlate of resistance; however, little is known about the role of these cells in Men Who Have Sex with Men (MSM) with high risk sexual behaviors. We evaluated a general overview of activation and effector features of NK cells of MSM co-cultured with LT CD4^+^ HIV^+^ in which MSM at high risk of HIV-1 infection (HR-MSM) exhibit higher capacity to eliminate infected cells, reduced percentages of CD69^+^ cells when compared to MSM at low risk of infection (LR-MSM). In addition, we found that, despite the lower levels of CD69^+^ NK cells on HR-MSM group, within this population, higher percentages of CD69^+^ IFN-γ^+^ and CD69^+^ NKG2D^+^ NK cells were found together with higher levels of RANTES and Granzyme B production with higher antiviral capacity, resulting in a lower concentration of p24 protein and p24^+^ CD4^+^ T cells. Altogether, this information suggests that NK cells of MSM could impact the capacity to face the viral infection.

## Introduction

The repeated contact with HIV-1 would suppose an infection. However, some individuals remain uninfected despite multiple high-risk exposures or repeated high‐risk behavior; this population is known as HESN. They are essential for the study of potential factors mediating natural resistance to HIV-1 infection^1^. HESN can be classified under three major groups: i) serodiscordant couples; ii) individuals with high-risk sexual behaviors, including commercial sex workers (CSW) and Men who have Sex with Men (MSM); iii) Individuals exposed non-sexually, including IDU, infants born to HIV-infected mothers, hemophiliacs, and others exposed to contaminated blood products^2^.

HESN allow the study of immunological and genetic features related to natural resistance to HIV infection. These features include CCR5 ∆32 mutation, immunological quiescence, HIV-1 specific IgA, HLA-KIR allele expression, HIV-1 specific cytotoxic lymphocytes (CTL), and production of soluble factors, among others^3^. Besides, NK cells have been also implicated in natural resistance to HIV-1 infection. The diverse functions of these cells are an important link between innate and adaptive immune responses. Moreover, NK cells can eliminate infected cells by secreting lytic granules containing perforin and granzymes, and are important producers of chemokines such as CCL3, CCL4, and CCL5, which are ligands for the HIV-1-co-receptor CCR5, thus inhibiting viral entry by blocking viral co-receptors^4^.

Almost two decades ago, Scott-Algara et al*.* reported in a HESN-IDU cohort, a higher cytotoxic activity of NK cells against K562 cells when compared to healthy controls and other IDU who seroconvert during the study. In addition, a higher percentage of positive CCL3, CCL4 and CCL5 and IFN-γ NK cells were found^5^. This work was the first evidence of the role of NK cells in HIV-1 resistance and since then, similar results have been published in several HESN cohorts^6–8^.

Recently, our research group reported that MSM at high-risk of acquiring HIV-1 infection exhibited a higher frequency of CD56^dim^/CD57 + /NKG2C^high^ NK cells than MSM at low risk. In addition, these individuals showed a higher cytotoxic capacity against K562 cells and a positive correlation between mRNA levels of IFN-γ and the percentage of CD57^+^/NKG2C^high^ NK cells^9^ highlighting the importance of this cellular population during the HIV-1 exposure. However, the functional and effector capacity of NK cells against autologous HIV-1 infected CD4^+^ T cells in MSM at high-risk of HIV-1 infection, has not been elucidated yet, which may clarify their potential in the phenomenon of natural resistance.

## Results

### MSM socio-demographic data

Twenty-two MSM fulfilled the inclusion criteria, with 11 individuals categorized for each group. Socio-demographic data are summarized in the Table 1. Statistical differences were found in the age of the individuals when compared LR-MSM and HR-MSM. The median of sexual partners in the last 3 months were 2 and 25 and the median of sexual partners in a lifetime were 27 and 1708 for LR-MSM and HR-MSM, respectively. In addition, the percentage of HIV-1 positive partners was higher in the HR-MSM group, denoting a higher sexual exposure to HIV-1 infection not only in the previous months of being included in the study but throughout their sexual life. In addition, no differences were found when comparing the percentage of unprotected sex in the last 3 months between both groups.

Finally, one individual belonging to HR-MSM group, had a heterozygous genotype for CCR5 *Δ32* mutation and is marked as ◈ symbol in the Figs. 2, 3, 4 and 6

**Table 1:** Socio-demographic data of MSM.

	HR-MSM	LR-MSM	*p* value
n	11	11	
Age, median	34	26	0.0199^a^
Sexual partners in last 3 months, median	25	2	< 0.0001^a^
% of unprotected sex in the last 3 months, median	50	39	ns
Sexual partners in a lifetime, median	1708	27	0.0019^a^
Frequency of STI*	86.44% (10/11)	55.34% (6/11)	< 0.0001^b^
Frequency of HIV-1 positive partners	64% (7/11)	45% (5/11)	0.0109^b^
Frequency of Δ 32 heterozygous	9% (1/11)	0% (0/11)	0.0032^b^

^a^ Mann–Whitney test ^b^ Fisher’s exact test *Sexually Transmitted Infections.

**Figure 1:**
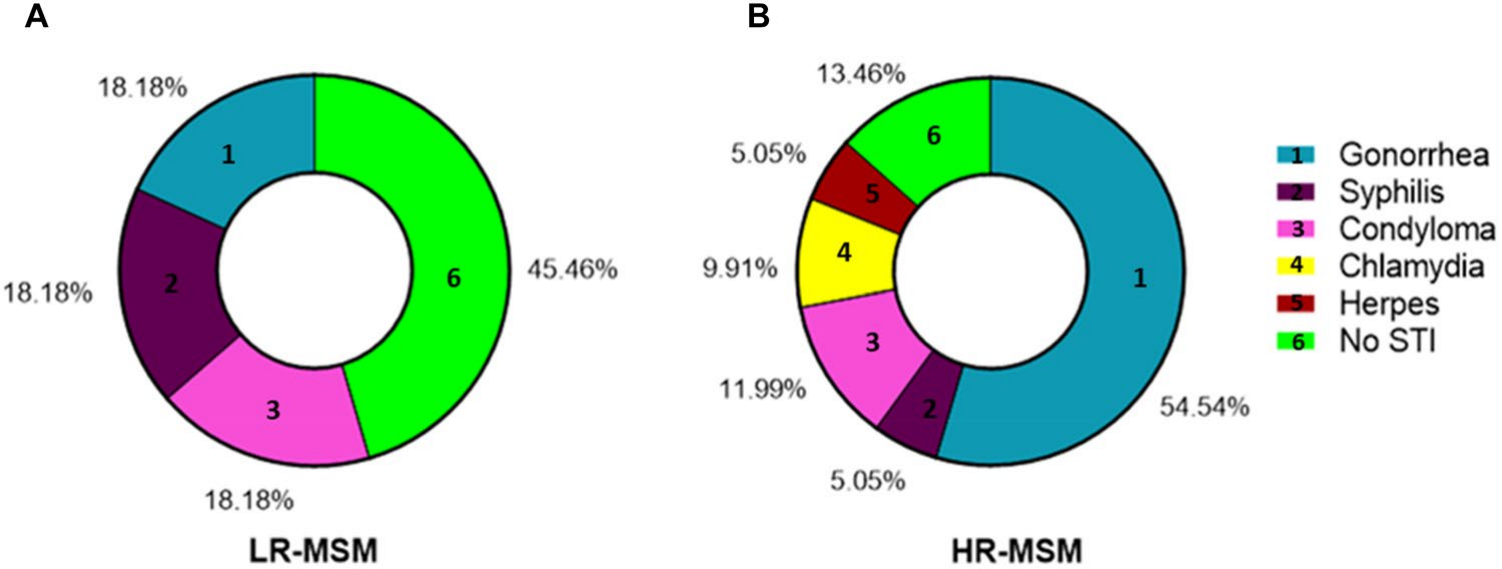
HR-MSM reported higher STI frequency compared to LR-MSM Distribution of STI in LR-MSM (**A**) and HR-MSM (**B**). The history of STI was self-reported by individuals in the study survey.

Among HR-MSM individuals, gonorrhea was the most common STI (Fig. 1)*.* In the LR-MSM population, a similar proportion of gonorrhea, syphilis and condyloma were found, 18.18% in each STI and, 45.46% of these individuals have not reported STIs (Fig. 1A). In contrast, 86.5% of HR-MSM reported STIs, including chlamydia and herpes, which were only found in this group (Fig. 1B).

### NK cells from HR-MSM showed higher antiviral activity compared to LR-MSM

**Figure 2:**
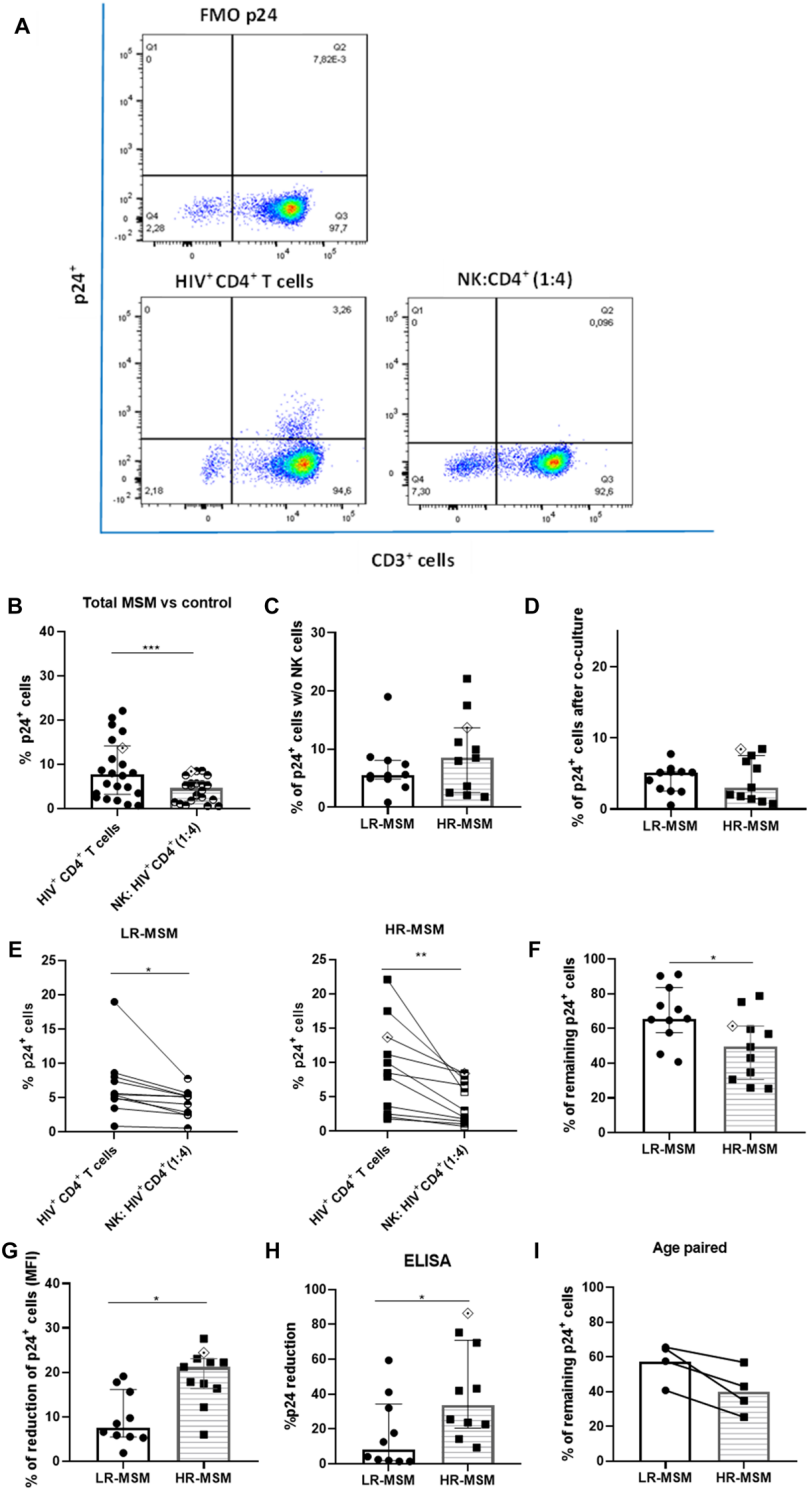
NK cells of HR-MSM showed a higher antiviral activity than LR-MSM. Representative gating strategy of p24^+^ cells evaluated in the co-cultures after 7 days of co-culture (FMO = Fluorescence Minus One control) (upper left panel), 80.000 HIV^+^ CD4^+^ T cells alone (lower left panel), and co-cultured NK with HIV^+^ CD4^+^ T cells in an effector:target ratio of 1:4 (lower right panel) (**A**). Percentage of p24^+^ cells in the co-cultures of all MSM individuals (LR-MSM and HR-MSM groups combined. Right bar) compared to infected control HIV^+^ CD4^+^ T cells (Left bar) (**B**). Percentage of p24^+^ cells measured in infected CD4^+^ T cells without NK cells (infection control) of LR-MSM and HR-MSM individuals (raw data) (**C**). Percentage of p24^+^ cells in LR-MSM and HR-MSM individuals after co-cultivation with NK cells at an effector target ratio of 1:4 (raw data) (**D**). Percentage of p24^+^ cells in the co-cultures of LR-MSM and HR-MSM compared to its respective infected control (**E**). Percentage of remaining p24^+^ CD4^+^ T cells in the co-cultures of LR-MSM and HR-MSM after co-cultivation with NK cells (**F**). Reduction of MFI of p24^+^ cells in the co-cultures expressed by percentage (**G**). Percentage of the p24 reduction measured by ELISA in the supernatants of the co-cultures after 7 days n:10 (**H**). Percentage of p24^+^ CD4^+^ T cells in the co-cultures of LR-MSM and HR-MSM age paired (**I**). (**B**–**F** and **G**): percentage of p24^+^ cells were measured by flow cytometry and normalized with infected control data. MFI (Mean Fluorescence Intensity). The results are shown as mean ± SD, n:11,11. Statistical evaluations were made with the Unpaired t-test and Mann-Whiney test. *p < 0.05 and ***p < 0.001. (**F**): remaining p24^+^ cells was calculated as the percentage of p24^+^ cells in co-cultured wells multiplied by 100. This result was divided by the percentage of p24^+^ cells in infected controls without NK cells. Heterozygous genotype for CCR5 *Δ32* mutation was marked as ◈ in the HR-MSM group.

After activation and HIV-1 infection of purified autologous CD4^+^ T cells, these cells were co-cultured with autologous NK cells in an effector-target ratio of 1:4. The antiviral activity of NK cells were measured after 7 days of co-culture through the evaluation of the p24^+^ cells by flow cytometry and, the detection of p24 protein in the supernatants by ELISA (Fig. 2). The representative gating strategy of the flow cytometry analysis is shown in Fig. 2A. These results showed a marked reduction in the percentage of p24^+^ cells in the co-culture with NK cells when both MSM groups were analyzed in combination against their respective infected control without NK cells (*p* = 0.0009) (Fig. 2B). Additionally, a tendency for a higher percentage of infection in the absence of NK cells (infection control) was observed in HR-MSM (8.5%) compared to LR-MSM individuals (5.5%). Still, no statistical differences were found (*p* = 0.438) (Fig. 2C). Remarkably, after co-cultivation with NK cells, the percentage of p24^+^ cells was lower in HR-MSM (3.05%) compared to LR-MSM individuals (5.13%); again, no statistical differences were found (Fig. 2D) (*p* = 0.9865).

In accordance, when LR-MSM and HR-MSM co-cultures were compared against their respective infected control, a higher percentage of reduction was found in HR-MSM (*p* = 0.0069) in comparison to the LR-MSM group (*p* = 0.0248) (Fig. 2E). In addition, when the percentage of p24^+^ cells was compared between LR-MSM and HR-MSM, a lower percentage of these cells were found in the HR-MSM group when compared to the percentage of remaining infected cells (49.2% on HR-MSM vs. 68% on LR-MSM) (*p* = 0.0217) (Fig. 2F). Moreover, the reduction of p24 expression measured by Mean Fluorescent Intensity (MFI) denoted a higher antiviral activity in HR-MSM (20.02) group compared to LR-MSM (7.523) (*p* = 0.0433) (Fig. 2G). Likewise, the p24 protein was measured in the supernatants of the co-cultures by ELISA and compared against their respective infected control (8 × 10^4^ LT CD4 + HIV^+^ without NK cells), a higher percentage of reduction of p24 protein was found in HR-MSM (33.6%) when compared to LR-MSM group (8.3%) (*p* = 0.0232) (Fig. 2H).

Finally, four LR-MSM and four HR-MSM were paired by age, and the percentage of p24^+^ cells was measured and compared for both groups. A tendency for a lower percentage of p24^+^ cells was observed in HR-MSM (37,9%) when compared to LR-MSM individuals (58,2%) (Fig. 2I); however, no statistical differences were found (*p* = 0.1250).

### HR-MSM showed a lower percentage of CD69^+^ in total NK cells than LR-MSM after co-culture

**Figure 3:**
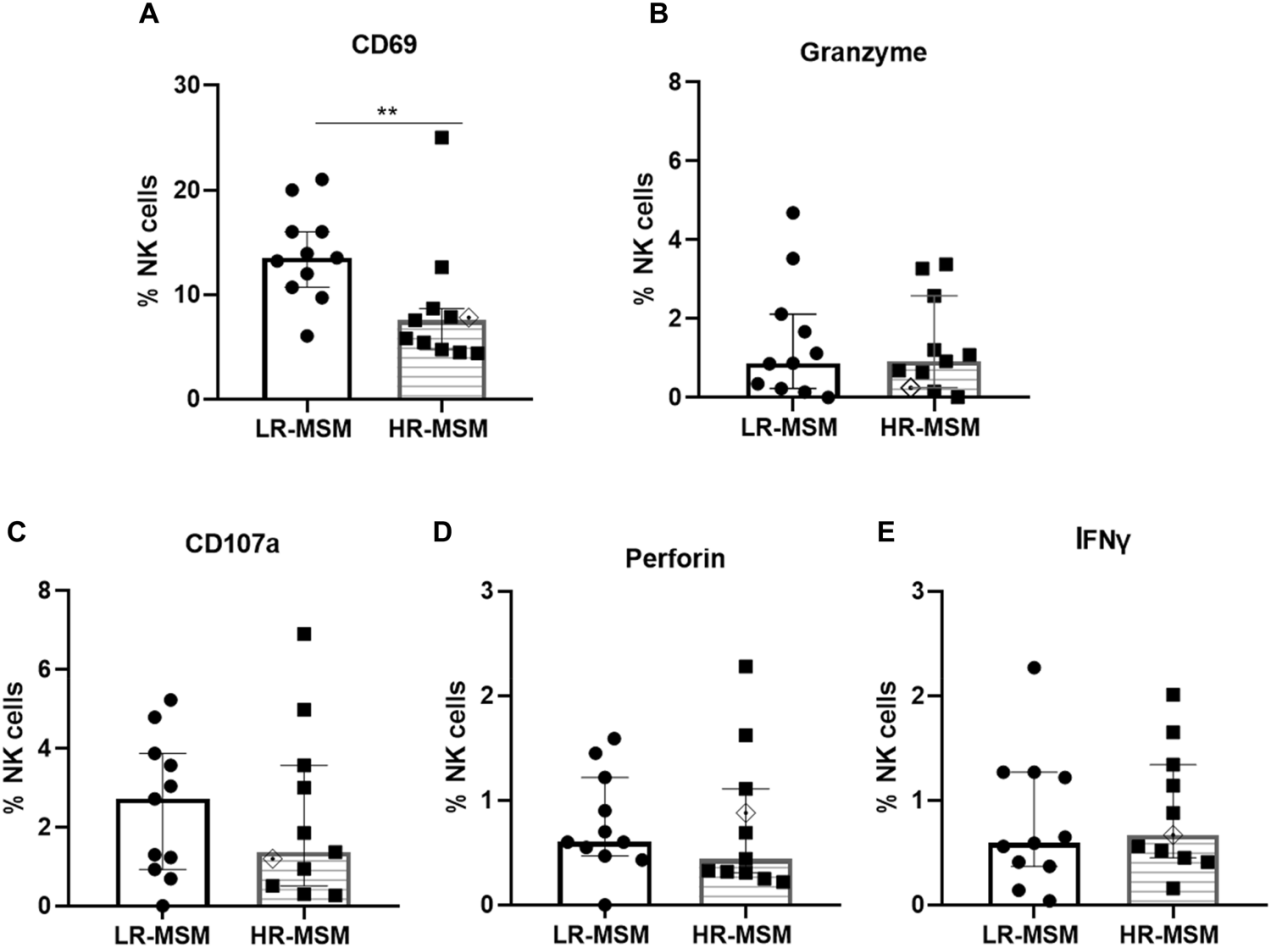
HR-MSM showed lower percentage of CD69^+^ NK cells but similar percentages of granzyme, CD107a, Perforin and IFN-γ when compared to LR-MSM. Percentage of NK cells expressing activation markers, evaluated in the co-cultures of LR-MSM and HR-MSM: CD69 (**A**); granzyme (**B**), CD107a (**C**), perforin (**D**) and IFN-γ (**E**). Bars represent the median with interquartile range. Statistical evaluations were made with Mann–Whitney U.**p < 0.01. Heterozygous genotype for CCR5 *Δ32* mutation was marked as ◈ in the HR-MSM group.

The percentage of activation markers was evaluated by flow cytometry on the NK cells co-cultivated with HIV-1-infected autologous CD4^+^ T cells after 12 h of the co-culture. A lower percentage of CD69^+^ NK cells was found in HR-MSM (7.5%) compared to LR-MSM (13.5%) (*p* = 0.005) (Fig. 3A). However, statistical differences were not reflected in the production of granzyme (1.41 vs. 1.28%) (*p* = 0.8286), CD107a (2.26 vs. 2.49%) (*p* = 0.7903), perforin (0.44 vs. 0.60%) (*p* = 0.5008), and IFN-γ (0.79 vs. 0.89%) (*p* = 0.7124) (Fig. 3C–E) production.

### Co-cultures of HR-MSM showed a higher percentage of CD69^+^ IFN-γ^+^ NK cells and high levels of IFN-γ production than LR-MSM

**Figure 4:**
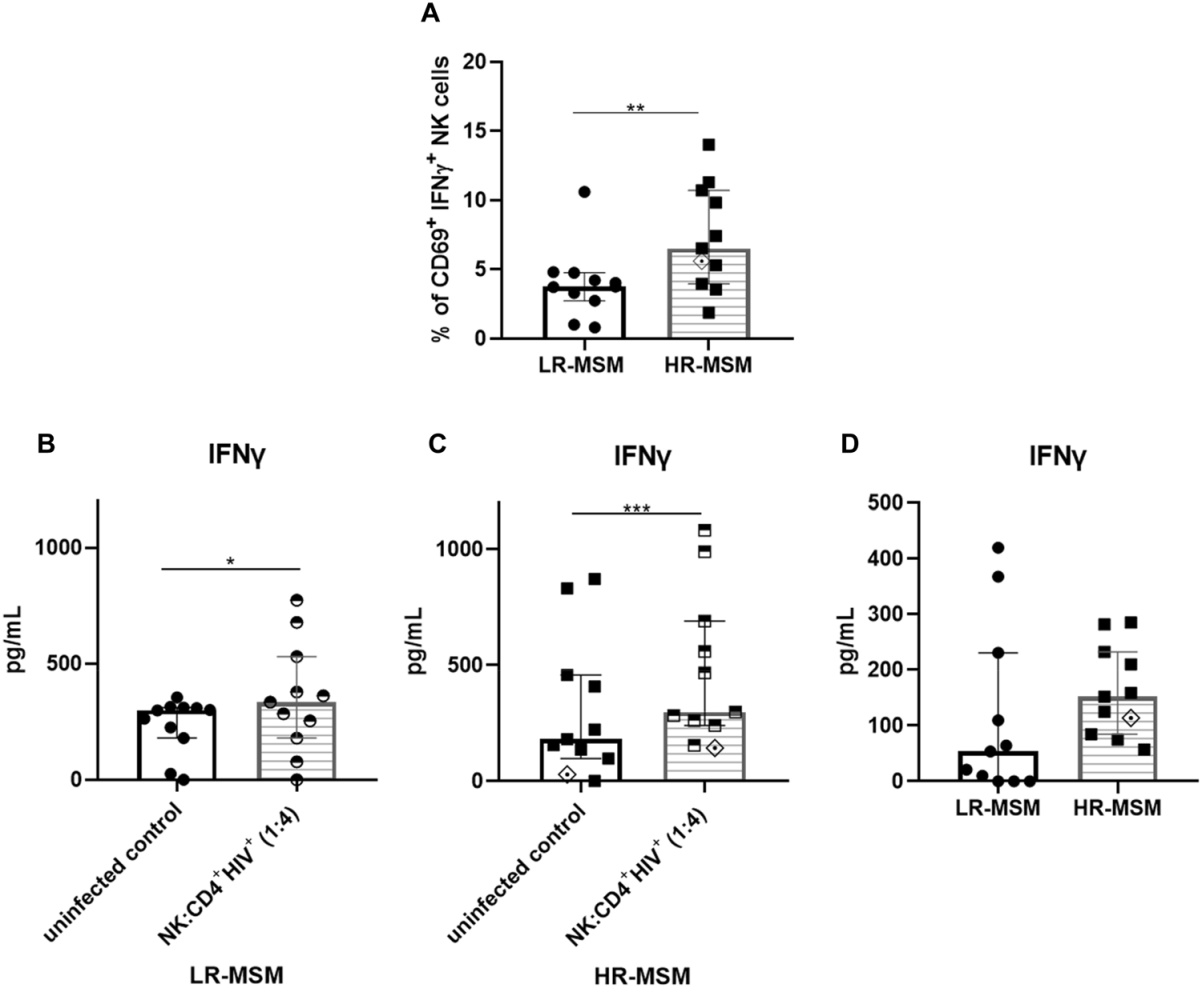
HR-MSM showed higher percentage of IFNγ^+^ activated NK cells and a tendency to higher production of IFN-γ compared to the LR-MSM. Percentage of CD69^+^ IFN-γ^+^ NK cells measured after 12 h of co-culture with CD4^+^ T cells infected with HIV (1:4) (**A**). Concentration of IFN-γ (pg/mL) in the supernatants of the co-cultures measured by CBA in LR-MSM comparing uninfected co-cultures with HIV-infected CD4^+^ T cells (**B**). Concentration of IFN-γ (pg/mL) in the supernatants of the HR-MSM co-cultures comparing uninfected control vs. HIV-infected CD4^+^ T cells (**C**). Concentration of IFN-γ (pg/mL) in the supernatants of the co-cultures of LR-MSM vs HR-MSM (**D**). The results are shown as mean ± SD, n:11,11. Statistical evaluations were made with the Mann-Whiney test and Wilcoxon. *p < 0.05, **p < 0.01 and ***p ≤ 0.001. Heterozygous genotype for CCR5 *Δ32* mutation was marked as ◈ in the HR-MSM group.

When analyzed alone, HR-MSM showed a lower percentage of CD69^+^ NK cells than LR-MSM (Fig. 3A)*.* However, the median of IFN-γ expression by CD69^+^ NK cells in HR-MSM (7.4%) were higher compared to the LR-MSM (3.9%) (*p* = 0.0026) (Fig. 4A). Suggesting that once activated, the NK cells from HR-MSM increase IFN-γ expression to exert its effector capacity.

Similarly, IFN-γ production was assessed by CBA in the supernatants of the co-cultures. Specifically, in comparison with the respective uninfected control, a higher significance was found in the HR-MSM group (*p* = 0.001) compared to the LR-MSM group (*p* = 0.0117) (Fig. 4B, C); however, no statistical differences were observed when LR-MSM and HR-MSM groups were compared (*p* = 0.0543) (Fig. 4D).

### CD69^+^ NK cells of HR-MSM exhibit functional differences when compared to LR-MSM

**Figure 5:**
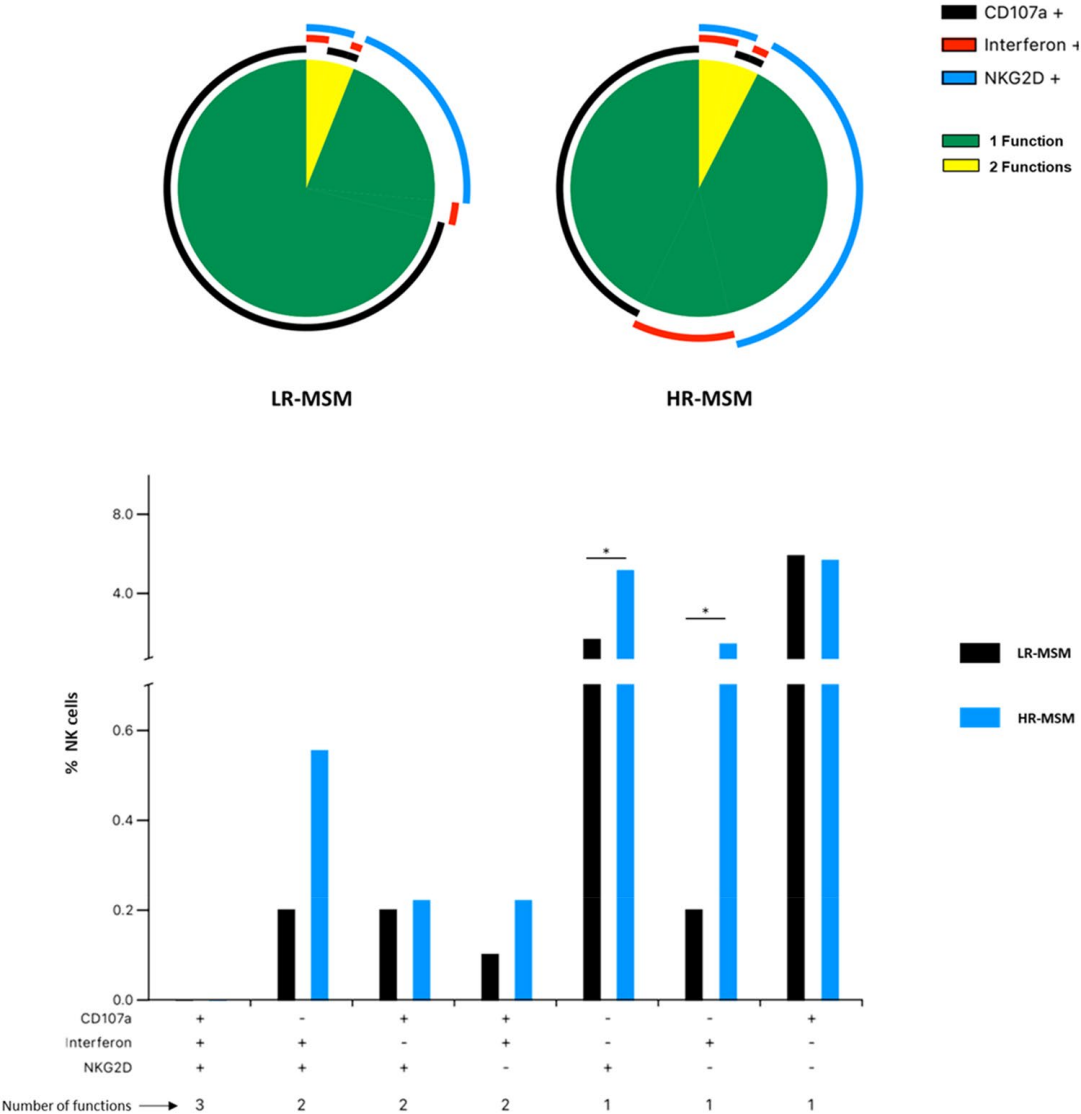
HR-MSM showed a higher frequency of NK cells with CD69^+^/IFN-γ^+^ and CD69^+^/NKG2D^+^ profiles. Polyfunctional profile analysis of NK cells of HR-MSM and LR-MSM after co-culture with CD4^+^ T cell HIV^+^ (1:4). The results are presented as mean, n:11,11 Statistical evaluations were made with Wilcoxon test *p < 0.05.

After the evaluation of the activation profile, we wanted to observe potential differences in the “activated” NK cells, briefly, starting from CD69^+^ subpopulation, the poly-functional profile of NK cells was evaluated after 12 h of co-culture by including the expression of NKG2D, IFN-γ and CD107a molecules (Fig. 5).

The results showed differences between both groups. Accordingly, CD69^+^/IFN-γ^+^ NK cells and CD69^+^/NKG2D^+^ NK cells were more frequent in HR-MSM than LR-MSM (*p* = 0.0114 for each analysis) (Fig. 5). Additionally, a tendency was observed in CD69^+^/IFN-γ^+^ /NKG2D^+^ and CD69^+^/IFN-γ^+^ /CD107a^+^ profiles with a higher frequency in the HR-MSM group; however, no statistical differences were observed.

### HR-MSM co-cultures showed higher Granzyme and RANTES levels than LR-MSM co-cultures

**Figure 6:**
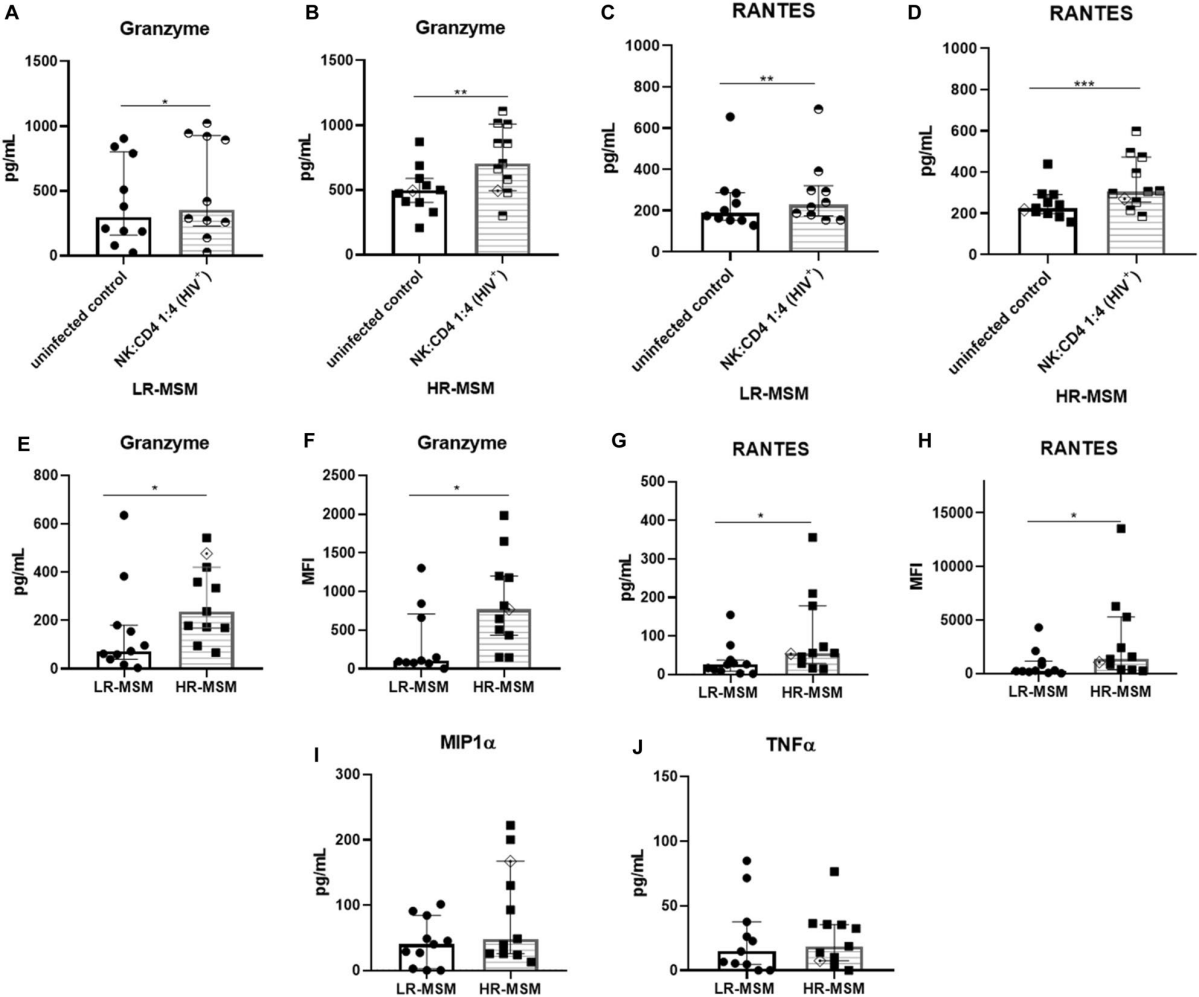
HR-MSM produces higher amounts of Granzyme and RANTES than LR-MSM. Effector molecules quantified in the supernatant by CBA after 12 h of co-culture. Concentration of Granzyme in the supernatants of uninfected and infected CD4^+^ T cells co-cultured with NK cells of LR-MSM (A) and HR-MSM (B) individuals. Concentration of RANTES in the supernatants of uninfected and infected CD4^+^ T cells co-cultured with NK cells of LR-MSM (**C**) and HR-MSM (**D**) individuals. Concentration of granzyme (**E**, **F**) and RANTES (**G**, **H**) in LR-MSM and HR-MSM expressed in pg/mL and Mean Fluorescence Intensity (MFI). MIP1-α and TNF-α concentration in the supernatants of the co-cultures of LR-MSM and HR-MSM (**I**–**J**). Graphs are presented as raw (**A**–**D**) and normalized data (**E**–**J**). The results are presented as median, n:11,11. Statistical evaluations were made with the Mann-Whiney test. *p < 0.05, **p < 0.01 and ***p ≤ 0.001. Heterozygous genotype for CCR5 *Δ32* mutation was marked as ◈ in the HR-MSM group.

The ability of NK cells to produce effector molecules was evaluated by CBA in the supernatants of the coculture after 12 h. Statistical differences were found in the concentration of granzyme and RANTES when NK cells were cocultured with uninfected and infected CD4^+^ T cells (Fig. 6A–D). In the LR-MSM group, a higher concentration of granzyme was found in the supernatants of NK cells cocultured with infected CD4^+^ T cells (353.9 pg/mL) compared to uninfected cocultures (295.5 pg/mL) (*p* = 0.0145) (Fig. 6A). Likewise, in the HR-MSM group, these statistical differences were also present when infected (705.7 pg/mL) and uninfected cocultures (495.5 pg/mL) were compared (*p* = 0.0020) (Fig. 6B). In agreement, RANTES concentration followed a similar tendency in LR-MSM and HR-MSM groups when infected (154.9 and 305.2 pg/mL, respectively) and uninfected (128.4 and 224.7 pg/mL, respectively) cocultures were compared (Fig. 6C, D) (*p* = 0.0020 and *p* = 0.0010, respectively).

Moreover, after normalization with uninfected control, these statistical differences were also observed in the concentration of granzyme for LR-MSM (72.81 pg/mL) compared to HR-MSM (237.70 pg/mL) (*p* = 0.040); this difference was also observed in the MFI, with a median of fluorescence of 100.8 in the case of LR-MSM and 770.6 in HR-MSM group (*p* = 0.0159) (Fig. 6E, F). Similarly, a lower concentration of RANTES was observed in the LR-MSM group (26.27 pg/mL) compared to the HR-MSM (55.73 pg/mL) (*p* = 0.0336) (Fig. 6G). Again, this difference was also observed in the MFI, with a median of 267.2 for LR-MSM and 1.352 for HR-MSM (*p* = 0.0192) (Fig. 6H). No statistical differences were found when MIP1-α (*p* = 0.0824) and TNF-α (*p* = 0.7621) production were compared for both groups (Fig. 6I, J).

### The number of sexual partners is correlated with reduction of p24^+^ cells and RANTES production

**Figure 7:**
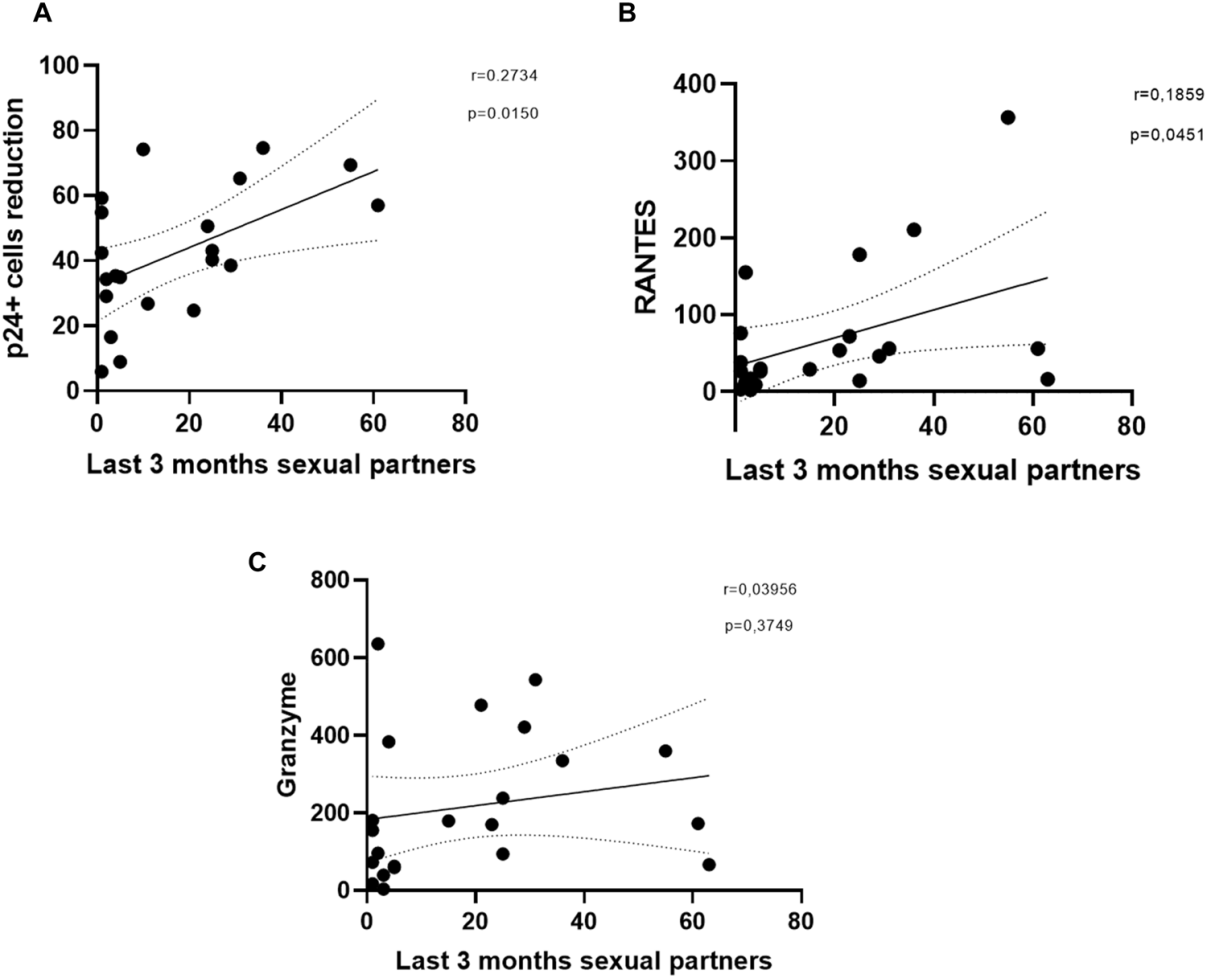
The number of sexual partners in the last three months is correlated with reduction of p24^+^ cells and RANTES production. Correlation of the number of sexual partners in the last three months in MSM individuals with the percentage of reduction of p24^+^ cells (**A**), production of RANTES (**B**) and granzyme (**C**) in the supernatants. Statistical evaluations were made with Spearman´s correlation test *p < 0.05.

The number of sexual partners in the last 3 months was a key criterion for categorizing LR-MSM and HR-MSM groups. For that reason, correlations between this parameter and the antiviral capacity of NK cells, in terms of their ability to produce granzyme and RANTES, were done. Remarkably, significant correlation was found with the percentage of p24^+^ cells reduction (r = 0.2734, *p* = 0.0150) (Fig. 7A), and RANTES production (r = 0.1859, *p* = 0.0451) (Fig. 7B). However, no correlation was found between granzyme production and the number of sexual partners in the last 3 months (Fig. 7C).

## Discussion

In recent years, the role of NK cells during HIV-1 infection has taken increasing importance. The antiviral activity of these cells is reflected in their ability to kill HIV-1 infected cells and the development of effector mechanisms that block viral entry, in addition to the enhancement of the immune response^10^. The experiments summarized in this work provide additional evidence on the protective function of NK cells in MSM individuals with a high-risk of acquiring HIV-1 infection. NK cells of HR-MSM exhibited a lower activation profile with higher antiviral capacity and, in accordance with previous reports in the same cohort^9^, increased production of effector molecules and a higher cytotoxic capacity were also observed.

Other studies, including MSM individuals, have reported and categorized an average of 3.2–6 sexual partners within the last 3 months as a high-risk behavior^11, 12^. The median of the sexual partners in the last 3 months in the current HR-MSM group was 25, and for the LR-MSM group, the median was 2, reflecting a higher sexual exposure in our HR-MSM group. In addition, our LR-MSM group is outside of the mentioned range, indicating lower exposure and risk practices, and this behavior is present not only in the number of sexual encounters in the last 3 months but in the number of sexual encounters in a lifetime in which, a considerable difference is observed when HR-MSM is compared to LR-MSM group. This information emphasizes the higher risk of acquiring HIV-1 infection in HR-MSM individuals. Subsequent studies on this cohort could help clarify the immunological advantages in the HESN populations when facing HIV-1. In our study, HR-MSM and LR-MSM groups shared biological factors and socio-cultural backgrounds that directly influence the risk of acquiring HIV-1 infection. These factors include but are not limited to anal exposure, sexual role (most of them versatile), condom use, ITS exposure, drug use, and reported blood transfusion procedures eliciting a similar exposure, which is visibly differentiated in the number of sexual partners characterized for this cohort^13^. Together, these characteristics support that the LR-MSM individuals are suitable controls for comparing the HR-MSM group evidence.

In agreement, MSM individuals were recruited near the downtown area of Medellín city, where they live, and frequent the same centers of diversity located in this area, both groups live under some stigma conditions such as social discrimination, sexism and lack of health services.

Moreover, in HR-MSM, a higher percentage of STIs (86,5%) were reported compared to LR-MSM (55,3%); the number of sexual partners in a lifetime could explain this difference, which is statistically different in both groups. There is a positive association between the number of STIs and the acquisition of HIV-1 infection^14^. Indeed, several reports have linked herpes and syphilis to augmenting the probability of HIV-1 infection due to an increase of CCR5^+^ T cells in the infected tissue^15^. Of notice, only one individual among both groups was heterozygous for the CCR5Δ32 mutation, and 21 individuals left were categorized as wild type, indicating that the most critical genetic mechanism of natural resistance against HIV-1 was absent in both groups. In addition, the percentage of unprotected sexual encounters in the HR-MSM group in the last 3 months was 50%, which is 11% higher than LR-MSM group (39%). This information, along with the fact that 64% of the HR-MSM group reported at least one HIV-1 positive sexual partner, evidence the high-risk of acquiring the infection for this population.

The HR-MSM group had a median of 34 years old and for LR-MSM group was 26. Some reports have concluded that the NK cell maturation process is highly age-dependent, in which young people showed higher numbers of CD56^bright^ cells compared to older people, which showed a higher frequency of CD56^dim^ expressing maturation markers as CD57^16^. Nevertheless, the differences in this phenotype can also be explained by accumulative exposure to different infections throughout the lifetime. For instance, in a study conducted in a Gambian population with a high frequency of HCMV (Human Cytomegalovirus) infection, it was reported that the percentage of terminally differentiated CD56^dim^ CD57^+^NKG2C^+^ NK cells in young children population, were near to 70% at the age of six, which is higher than the median of the frequency of this fully mature phenotype reported in European population which is around 50%^17^. In addition, there is also evidence that the increase of the CD56^dim^ population in the circulation of elderly individuals does not correlate with an increase in overall cytotoxicity of NK cells^18^, suggesting that despite age is involved in the stimulation and “training” of NK cells, there could be other mechanisms impacting the NK cells functionality.

We reported lower levels of p24^+^ CD4^+^ T cells in the HR-MSM and LR-MSM co-cultures compared to their respective controls, and these results were validated when compared to the levels of viral p24 protein in the co-culture supernatants measured by ELISA. Of note, despite the percentages of p24^+^ cells in the infection control were slightly higher in HR-MSM, no statistical differences were found when this percentage were compared to LR-MSM group. The mentioned differences could be due to a higher “basal” activation state in the CD4^+^ T cells of HR-MSM group, which have reported a higher percentage of STI’s.

Remarkably, both HR-MSM and LR-MSM individuals, showed to be functional eliminating infected CD4^+^ T cells. Although this capacity seems to be higher in the HR-MSM group when the reduction of p24^+^ cells is compared. The potential of NK cells for clearance of infected cells has been of particular interest in the HIV-1 context. Richard et al*.* described that HIV-1 infection could induce the up-regulation of specific ligands for NKG2D receptor of NK cells, such as ULBP proteins induced by HIV-1 accessory protein Vpr, enhancing NK cell-preference lysis of infected cells^19^. Other mechanisms related to the HIV-1 antiviral role of NK cells include the production of β-chemokines, ADCC, and IFN-γ production, which influence the adaptive immunological response^20^. Notably, this antiviral activity against HIV of NK cells can be enhanced through stimuli with IL-15, promoting the elimination of latently infected CD4^+^ T cells in vitro once they were induced to emerge from latency^21^. In the present work, this cytokine was used overnight, (20 ng/mL), to maintain the NK cells viability before the co-culture. However, statistical differences between HR-MSM and LR-MSM groups were found, indicating that, despite being primed under the same conditions, NK cells of HR-MSM individuals had a higher antiviral capacity. Remarkably, the percentage of NK cells used in the co-cultures of the present study corresponds to 25% and the 75% left corresponded to infected CD4^+^ T cells. This Effector-Target ratio (1:4) matches with the mean frequency of NK cells (15.8%) and CD4^+^ T cells (43%) reported in peripheral blood of a Latin American cohort^22^. In addition, a tendency for a lower percentage of p24^+^ cells were observed in HR-MSM compared to LR-MSM even after being paired by age, indicating that this parameter is not a direct correlate of the differences found in the antiviral capacity of NK cells of the MSM population on this study. Altogether, this evidence indicates that a higher antiviral capacity of HR-MSM NK cells could have a biological impact on the HIV-1 resistance observed in these individuals.

Some markers associated with NK cell activation were analyzed as well. Our results showed a lower percentage of CD69^+^ NK cells in HR-MSM than LR-MSM group; however, no statistical differences were found when perforin, granzyme, IFN-γ and CD107a were compared in the total percentages of NK cells. This evidence was described in other HESN individuals in which low percentages of CD69^+^ NK cells were found in the peripheral blood of CSW-HESN women^23^. Moreover, a reduced expression of this marker in CD4^+^ T cells has been historically associated with quiescence phenotype, which is characterized by lower HIV target cell availability and susceptibility^24^. Remarkably, other markers associated with NK cell activation did not show statistical differences, indicating that a lower frequency of CD69^+^ NK cells is not a synonym of activation impairment. In contrast, other reports have suggested that an increased expression of CD56^dim^ CD69^+^ NK cells prevail in HESN individuals^25^. However, recent evidence suggests that the differentiation into an adaptive phenotype such as NKG2C^+^ NK cells seem to be linked to the expression of HLA-DR, unlike the expression of other activation markers such as CD69 and CD25^26^. In agreement, more than two decades ago, it was demonstrated that the terminally differentiated CD56^dim^ NK cells population in older people exhibits higher levels of HLA-DR and CD95, along with a decrease of the CD69 marker^27^, also found in lower percentages in our study. We hypothesize that NK cells from HR-MSM exhibit a lower activation profile and possibly higher expression of the NKG2C marker. This is supported by previously reported evidence from our research group in which HR-MSM had a higher percentage of CD56^dim^ NK cells and a higher frequency of adaptive CD57^+^/NKG2C^high^ cells NK when compared to LR-MSM^9^ group. Likewise, this mature phenotype has been related to a better cytotoxic activity linked to the enhanced capacity to eliminate infected CD4^+^ T cells.

The effects of IFN-γ in pro-inflammatory responses, immune activation, and antiviral activity have converted this cytokine into an interesting marker to evaluate immunological capability against HIV. In the case of HESN individuals, a higher percentage of IFN-γ^+^ NK cells were reported compared to uninfected and HIV^+^ controls after the stimulation with PMA/Ionomycin^8^. Another study in uninfected infants born to HIV-1 infected mothers showed that HIV-*gag* specific IFN-γ cellular response detected in breast milk was associated with decreased infant HIV-1 infection in HESN infants^28^. As mentioned before, a lower frequency of CD69^+^ NK cells were found in HR-MSM than LR-MSM. Despite this, a higher percentage of CD69^+^ IFN-γ^+^ NK cells were found in HR-MSM compared to LR-MSM. Although, no statistical differences were found in the production of IFN-γ among LR-MSM and HR-MSM; when IFN-γ levels were compared against their respective uninfected control, a higher statistical significance was found in HR-MSM group; suggesting wide biological differences in the basal production or regulation of IFN-γ among all individuals enrolled in this study.

The functional analysis of CD69^+^ NK cells showed two different NK cell populations, CD69^+^/IFNγ^+^ and CD69^+^/NKG2D^+^ NK cells with higher frequency in HR-MSM. In the MSM-HESN population, little is known about the co-expression of these markers; however, they have been linked to HIV-1 antiviral activity in other HESN cohorts^29^. Interestingly, in HIV-1 infected individuals, *Nabatanzi *et al*.* reported an atypical activation pattern in NK cells among ART-treated individuals, with a higher CD69^+^ expression and a lower expression of IFN-γ, NKG2D, and granzyme B. In contrast, we reported a lower frequency of CD69^+^ NK cells in HR-MSM individuals with an augmented functional capacity, reflected in the CD69^+^/IFN-γ^+^ and CD69^+^/NKG2D^+^ expression along with a tendency for higher CD69^+^/IFNγ^+^ /NKG2D^+^ NK population. Of note, the functional recovery of NK cells is a topic of particular interest in HIV-positive individuals^30, 31^, as well as in the natural resistance mechanisms against HIV-1, in which IFN-γ^+^ and NKG2D^+^ expression seem to be involved^5, 32^. In the present study, the total percentages of NKG2D + NK cells were not measured in the fresh blood of MSM due to limitations in the number of acquired cells. However, this limitation could be approached in future studies and hopefully help us establish a correlation between this marker and HIV-1 natural resistance in MSM individuals.

Furthermore, studies performed in a Colombian HESN cohort have shown a higher expression of granzyme by PBMCs, in a basal state and even after 7 days of in vitro infection with HIV-1, compared to HIV-positive individuals and uninfected controls^33^. In addition, studies performed on elite controllers have linked higher levels of granzyme B with the capacity to control viral replication^34^ highlighting the role of this protease. In HESN cohorts, recent reports have found high levels of granzyme B, TNF-α, and IFN-γ produced by NK cells in cervicovaginal lavages compared to unexposed-healthy women^29^. Our findings also showed a higher concentration of granzyme B in the supernatants of the co-cultures of HR-MSM, with a tendency to higher levels of TNF-α and IFN-γ, which could be linked to differences in the percentages of p24^+^ CD4^+^T cells observed, suggesting that granzyme B could be an important natural resistance mechanism to HIV-1 infection.

The evaluation of molecules with antiviral activity in supernatants of the co-cultures by CBA showed that RANTES (CCL5) was produced in a higher concentration for HR-MSM than LR-MSM. Interestingly, this β-chemokine is associated with inhibiting HIV-1 entry through binding to the CCR5 coreceptor, interrupting the interaction with the HIV envelope glycoprotein gp120^35^. Similarly, RANTES was reported to be in higher percentages on NK cells of peripheral blood of HESN-IDU. Indeed, this difference remains after co-culture with K562 cells compared to seropositive controls before and after infection^5^. This evidence indicates that a higher frequency of CCL5^+^ NK cells could be involved in natural resistance^36^. Likewise, higher production of RANTES has been correlated with protection against HIV infection in different biological compartments such as peripheral blood, saliva, or genital mucosa among different cohorts of HESN around the world^37, 38^. These findings suggest that higher production of RANTES by NK cells could reduce the HIV-1 infected CD4^+^ T cells, which is reflected in the percentages of p24^+^ CD4 T cells in the evaluated co-cultures of HR-MSM individuals.

Finally, p24 levels reduction and RANTES expression were correlated to the magnitude of sexual exposure in the last 3 months, suggesting that higher exposure to HIV-1 infection in a recent period in HR-MSM individuals could be involved in a “trained” state of NK cells, allowing these cells to respond more robustly to different microbial infection^39^. In conclusion, HIV-1 natural resistance is a multifactorial state based on intrinsic biological differences with multiple edges. For that reason, evaluating the effector capacity of NK cells is only a step forward in deciphering natural resistance to HIV-1 in some MSM individuals.

## Methods

### Ethical approval and informed consent

The study was performed according to the principles of the declaration of Helsinki and approved by the ethics committee from the Universidad de Antioquia (Act No.007, May 22, 2014). All the individuals enrolled in the present study provided signed informed consent forms.

### Study population

A total of 22 MSM from Medellin-Colombia were recruited from a cohort previously established^13^. MSM were divided into two groups: i) MSM at high-risk of infection: MSM with more than 15 different sexual partners in the last 3 months with reported unprotected sexual intercourse (HR-MSM), and ii) MSM at low risk of infection: MSM with less than 4 different sexual partners in the last 3 months with reported unprotected sexual intercourse (LR-MSM). MSM younger than 18 years of age, positive for HIV 1/2 rapid test (SD BIOLINE), positive for HIV proviral DNA PCR or homozygous for CCR5 Δ32 mutation were excluded.

A survey for risk behavior was applied to all individuals and, 50 mL of peripheral blood were taken with a disposable syringe with EDTA.

### NK cells anti-HIV activity

Peripheral blood mononuclear cells (PBMCs) were isolated through density gradient using Ficoll-Histopaque (Sigma-Aldrich, St. Louis, MO, USA) by centrifugation at 400 g for 30 min. PBMCs were washed with PBS 1X to eliminate platelets. After, cells were counted and cryopreserved until they were used.

PBMCs were thawed and let in culture in RPMI with 10% fetal bovine serum (FBS) (Gibco, Grand Island, NY) for 24 h before each experiment. NK and CD4^+^ T cells were isolated in parallel from each donor by negative selection (Miltenyi Biotec, Bergisch Gladbach, Germany). The NK cell isolating antibody cocktail included monoclonal antibodies against CD3, CD4, CD14,CD19,CD20, CD36, CD123, HLA-DR, and glycophorin. The isolating antibody cocktail for CD4^+^ T included CD8, CD14, CD15, CD16, CD19, CD36, CD56, CD123, TCR γ/δ, and CD235a (Glycophorin A). Briefly, PBMCs designated to CD4^+^ T isolation were stimulated for 48 h with 8 μg/mL of Phytohemagglutinin- PHA (Sigma-Aldrich, St. Louis, MO) and 50 UI/mL IL-2 (Peprotech, Rocky Hill, CT). After stimulation, CD4^+^ T cells were isolated using the previously mentioned antibody cocktail and infected by spinoculation with 1 ng of p24/million cells of HIV-1Ba-L (donated by AIDS Research and Reference Reagent Program, Division of AIDS) for 90 min at 700×g. After spinoculation, cells were washed twice with PBS 1 × to remove free virions.

Then, 8 × 10^4^ HIV-1BaL-infected CD4^+^ T cells and 2 × 10^4^ NK cells were co-cultured and plated in duplicate in a 96-V bottom well plate at an Effector-Target ratio of 1:4 (one NK cell per four infected CD4^+^ T cells) and left in culture for 7 days in RPMI plus 10% FBS supplemented with 15 UI/mL of IL-2. Prior to co-cultivation, NK cells were pre-treated with 20 ng/mL of IL-15 overnight. Viral production was assessed in the supernatant by HIV-1 p24 ELISA (Xpressbio, Ballenger Creek, Maryland, USA).

In addition, antiviral capacity of NK cells was also assessed by measuring intracellular p24 in CD4^+^ T cells, after co-cultures. Briefly, co-cultured cells were stained with antibodies against CD56 (CMSS; Thermo Scientific, Wilmington, DE, USA) and CD3 (UCHT1; Thermo Scientific, Wilmington, DE, USA) for 20 min in the dark. Later, cells were treated with Foxp3 / Transcription Factor Staining Buffer Set (Thermo Scientific, Wilmington, DE, USA) according to the manufacturer´s guidelines to permeabilize them. After were stained with anti CD4 (RPA-T4; BD Biosciences, San Jose, CA, USA) and anti p24 (KC57; Beckman Coulter, Pasadena, CA, USA). After infection, downregulation of CD4 + was observed on co-cultured lymphocytes, for that reason, to avoid underestimation, the percentage of p24^+^ CD3^+^ cells were measured instead (Supplementary Fig. 1,2). Data were analyzed using FlowJo version 10.5.3 (FlowJo, LLC, Oregon, USA), and normalized with infected CD4^+^ T cells in absence of NK cells.

### Evaluation of NK cell activation

NK cell activation was assessed by flow cytometry at 12 h after the co-culture. Prior staining, 6 µg/mL of Brefeldin A, 2 mM of Monensin (both from Thermo Scientific, Wilmington, DE, USA) and 1µL of anti-CD107a (BD Biosciences, San Jose, CA, USA) were added to the culture and incubated at 37 °C, 5% CO_2._ Then, cells were stained with antibodies against CD56 (CMSS); NKG2D (1D11); and CD69 (FN50); (all of them from Thermo Scientific, Wilmington, DE, USA), and incubated for 20 min in the dark. In addition, cells were also treated with Foxp3 / Transcription Factor Staining Buffer Set and then stained with CD3 (UCHT1; Thermo Scientific, Wilmington, DE, USA), IFN-γ (4S.B3, Biolegend), Granzyme B (BD Biosciences, San Jose, CA, USA) and Perforin (δG9; BD Biosciences, San Jose, CA, USA). Data were analyzed using FlowJo version 10.5.3 (FlowJo, LLC, Oregon, USA). Data were normalized with NK cells co-cultured with uninfected CD4^+^ T cells and SPICE platform were used to carry out NK poly-functionality tests.

### Quantification of antiviral molecules by Cytometric Bead Assay (CBA)

Similar to the evaluation of NK cell activation, supernatants of co-cultured cells were collected, however they were obtained from independent wells and stored at − 80 °C until they were used. Supernatants were thawed at 4 °C right before running the CBA assay. The panel for the CBA flex set included: TNF-α, Granzyme, IFN-γ, MIP-1α and RANTES (BD Biosciences, San Jose, CA, USA). The CBA assay was done according to the manufacturer´s instructions. The beads complex was acquired using LS Fortessa (BD Biosciences, San Jose, CA, USA). Obtained data were normalized with NK cells co-cultured with uninfected CD4^+^ T cells and analyzed using FlowJo version 10.5.3 (FlowJo, LLC, Oregon, USA).

### Statistical analysis

HR-MSM and LR-MSM data were compared with Mann–Whitney *U, Wilcoxon,* or Student’s *t*-test, depending on the bivariate normality assumption and according to the Shapiro–Wilk normality test. A *p*-value < 0.05 was considered statistically significant. Statistical tests were performed using GraphPad Prism Software version 8.01 and SPICE platform were used for NK poly-functionality tests. Only data with a representation higher than 0.1% were included for this analysis.

Multivariate analyses were not initially considered because our main objective was focused on the effector capacity of NK cells. However, we know that some variables, such as age and biological/socio-cultural background, could affect the NK cells function. For that reason, additional analysis including individuals with similar age and backgrounds, in both groups were done.

### Supplementary Information

Supplementary Information.

## Data Availability

The data that support all the findings of this study are available from the corresponding author upon request.
