# Supplementary material for: NK cells from Men Who Have Sex with Men at high risk for HIV-1 infection exhibit higher effector capacity
Source: Sci Rep. 2023 Oct 5;13:16766. doi: 10.1038/s41598-023-44054-1 (PMC10556081; doi:10.1038/s41598-023-44054-1)
Supplement: Supplementary file 1 — Supplementary Information. [file 41598_2023_44054_MOESM1_ESM.doc]

**Supplementary information**


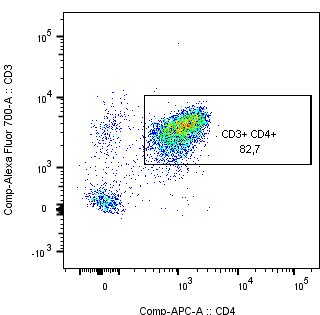

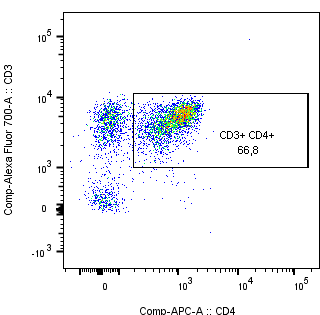


**CD3^+^**

**CD4^+^**

**A**

**B**

**Supplementary figure 1**: **CD4 Expression is downregulated after infection with HIV-1 *in vitro*. A**. Percentage of CD3^+^ CD4^+^ cells in non-infected co-cultures after seven days. **B**. Percentage of CD3^+^ CD4^+^ cells in infected co-cultures after seven days.

**p24^+^**

**CD4^+^**

**CD3^+^**

**p24^+^**


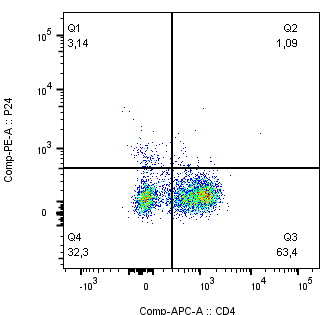


**A**

**B**


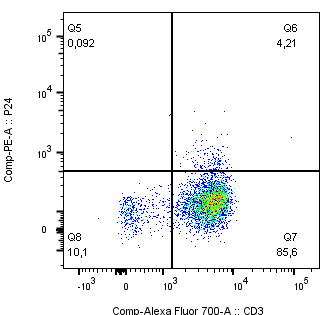


**Supplementary figure 2**: **Percentages of p24+ cells could be underestimated due to downregulation of CD4 marker. A**. Percentage of p24^+^ cells measured on CD4^+^ T cells after seven days of infection. **B**. Percentage of p24^+^ cells measured on CD3^+^ cells after seven days of infection.


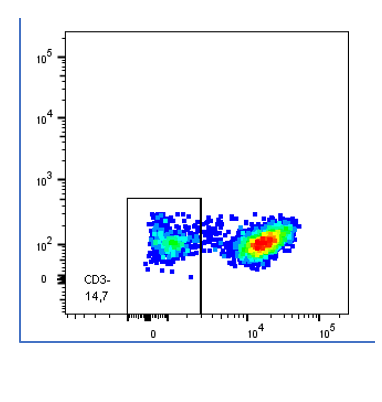

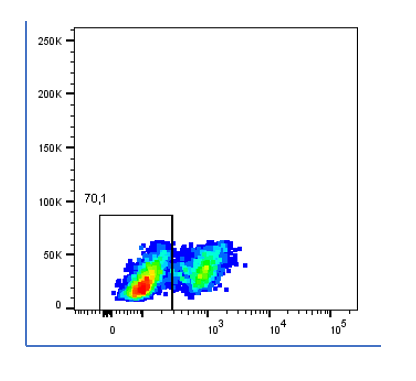


**VIABILITY**

**SSC-A**

**VIABILITY**

**CD3**


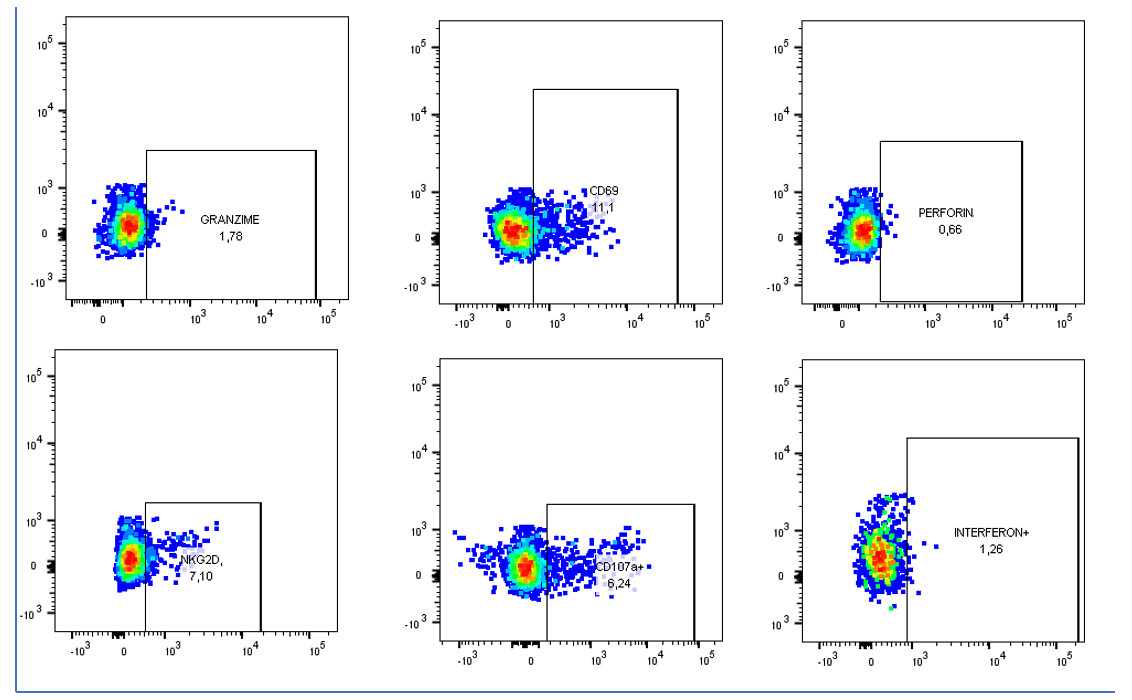


**CD3-**

**Supplementary figure 3**: **Gating Strategy of NK activation markers**. Gating strategy of activation markers evaluated after co-culture; Granzyme, CD69, Perforin, NKG2D, CD107a and Interferon. NK cells were selected after viability marker (left upper panel) in the CD3- region.
